# Phylogenetic characterization of norovirus strains detected from sporadic gastroenteritis in Seoul during 2014–2016

**DOI:** 10.1186/s13099-018-0263-8

**Published:** 2018-08-27

**Authors:** Young Eun Kim, Miok Song, Jaein Lee, Hyun Jung Seung, Eun-Young Kwon, Jinkyung Yu, Youngok Hwang, Taeho Yoon, Tae Jun Park, In Kyoung Lim

**Affiliations:** 1 Department of Infectious Disease Research, Seoul Metropolitan Government Research Institute of Public Health and Environment, 30 Janggunmaeul 3-gil, Gwacheon, 13818 Republic of Korea; 2 Department of Biochemistry and Molecular Biology, Ajou University School of Medicine and Graduate School of Medicine, 164 Worldcup-ro, Yeongtong-gu, Suwon, 16499 Republic of Korea

**Keywords:** Norovirus-GII.4, -GII.17, -GII.3 surveillance, Virus epidemiology, Phylogenetic analysis, Molecular characterization, Sporadic AGE (acute gastroenteritis)

## Abstract

**Background:**

Phylogenetic analysis of norovirus (NoV) is efficient for tracking NoV transmission. To determine the widespread NoV strains in Seoul, we conducted an extensive phylogenetic characterization of NoV-positives from 1659 diarrheal specimens collected in 2014–2016 for the Seoul NoV-surveillance.

**Results:**

When the large numbers of NoV partial VP1 genome sequences were analyzed in acute gastroenteritis patients along with the phylogenetic characterization, we could identify molecular epidemiologic patterns based on the genetic characteristics of sporadic NoV strains circulating in Seoul, which could provide a detailed description of the genome-wide and community-wide NoV evolution in each genotype. The average NoV detection rate in our study period was 16.34% that was increased by 7.44% from 13.17% in 2014 to 20.61% in 2016. Prevalence of NoV GI and GII was 4.43% and 93.36%, respectively, and the GII.4, GII.17, and GII.3 were found to be the major type among 17 genotypes of NoV. The most prevalent one was GII.4 (50.92%) that was followed by GII.17 (18.08%) and GII.3 (9.96%). According to an extensive phylogenetic analysis based on partial VP1 sequences of 1008 NoV (276 sporadic, 518 outbreak and 214 reference), pandemic strains of GII.17, GII.4 and GII.3 have emerged in succession during the 2014-2016 Seoul NoV-surveillance. GII.17 emerged as GII.17|Kawasaki323 in 2014, and became the predominant genotype in 2015 with GII.17|2014_Kawasaki lineages (CUHK-NS-616/Kawasaki308). The formerly predominant GII.4 remained high-level with GII.4|2012_Sydney in 2014 and internally replaced to GII.4|2016_Kawasaki194 lineage (NOR-2565/NOR-2558/OH16002) that caused the sporadic NoV explosion since December 2015. Sporadically prevalent GII.3|Hu/Aichio334-13/2013 failed to develop any outbreaks, whereas sporadic GII.3|Hu/3-28/2015/HNZZ/CHN caused heavy outbreaks in Seoul without preparation time since November 2016.

**Conclusions:**

This is the first extensive phylogenetic study revealing the important events of NoV strains circulating in Seoul. Particularly, our study period from 2014 to 2016 was very dynamic with the emergences of the three main NoV strains (GII.17|2014_Kawasaki, GII.4|2016_Kawasaki194 and GII.3|Hu/3-28/2015/HNZZ/CHN) every year. We are sure that it is hard to detect above findings by simple conventional analysis. Our present study reports a future paradigm of the NoV molecular epidemiology, which might be highly valuable to track new strains and predict oncoming outbreaks.

**Electronic supplementary material:**

The online version of this article (10.1186/s13099-018-0263-8) contains supplementary material, which is available to authorized users.

## Background

Acute gastroenteritis (AGE) causes one of the major public health problems [1], and NoV has been reported as the most common cause of AGE [2]. NoV is a non-enveloped, positive-sense, single-stranded RNA virus with a linear genome (7.5–7.7 kb), which belongs to the family Caliciviridae with three open reading frames (ORFs) encoding nine structural and nonstructural proteins [3–5]. ORF1 encodes nonstructural proteins such as NTPase, protease, and RNA-dependent RNA polymerase (RdRp). ORF2 overlaps ORF1 by a short region and encodes the major capsid protein, VP1. ORF3 encodes the minor capsid protein, VP2 [6]. NoVs are highly diverse and currently sub-divided into six genomic groups (GI/GII/GIII/GIV/GV/GVI) with more than 40 genotypes based on their VP1 sequences [7, 8].

It has been reported that NoV caused at least six pandemics of AGE (defined as taking place on at least three continents over a similar time-frame) since 1995; 1995–1996 (GII.4|US95_96), 2002–2003 (GII.4|Farmington Hills), 2004–2005 (GII.4|Hunter), 2006–2007 (GII.4|2006a_Yerseke and GII.4|2006b_Den Haag), 2009–2010 (GII.4|New Orleans) and 2012–2013 (GII.4|Sydney) [8]. NoV exhibits over 40 genotypes co-circulating within the population, however, GII.4 has emerged only as novel variants about every 2–4 years, massive outbreaks, and pandemics [9]. Like influenza virus, population immunity may drive the evolution of NoV and the emergences of its new variants [10], which undergoes genetic and antigenic evolution through accumulation of point mutations and intra- and inter-genotype recombinations [11].

As awareness and knowledge about the growth of Seoul NoV epidemiology, the question has been raised how to effectively track the emergence of new NoV strains, and how to monitor the spread of them. We, therefore, tried to create a more extensive phylogenetic characterization of our data obtained from the Seoul NoV surveillance. This NoV-surveillance system aimed at controlling the spread of future NoV outbreaks by monitoring the circulating strains. Here, we presented the widespread- and newly emerged-NoV strains in Seoul and tried to characterize their molecular epidemiology in the 2014–2016 Seoul surveillance. During the 3-year study period, we have observed novel epidemic strains found in global distribution, however, their sub-lineages showed different scale and impact in distribution or prevalence along with co-circulating strains in Seoul. Here, we also reported sporadic strains developed into outbreaks in our NoV-surveillance system. Total 1008 sequences were analyzed phylogenetically in the five different NoV models (GI/GII.4/GII.17/GII.3/other types of GII).

## Methods

### Ethics statement

All the processes from sample collection to diagnosis of NoV were followed by National Norovirus Surveillance System in Korea (K-CaliciNet) and the “Guideline for water and foodborne diseases prevention and control” [12] under the Korean “Enforcement Regulations of the Infectious Disease Control and Prevention Act”. All data were handled based on the Korea Centers for Disease Control and Prevention (KCDC) regulations. Present study was carried out after the diagnosis of NoV as an anonymous epidemiological data and a phylogenetic characterization of NoV sequences. According to the Human Subjects Institutional Review Board (IRB) of Korea National Institute for Bioethics Policy, our present study is excluded from the subject to deliberation.

### Specimens and diagnosis

**Fig. 1:**
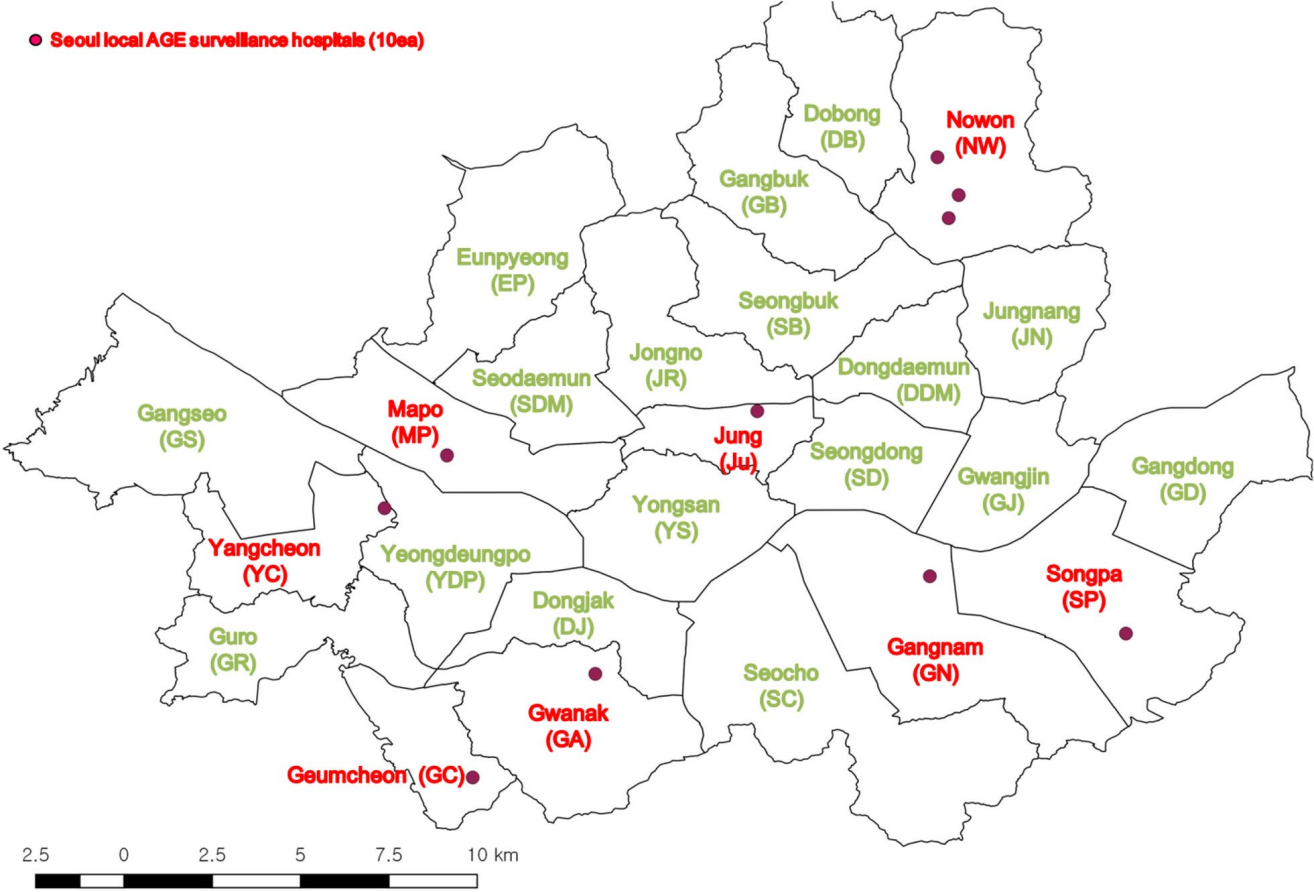
A map representing the 25 administrative district-Gu-in Seoul, Korea. The solid circles in red represent the 10 local hospitals participated in the AGE surveillance in Seoul

Diarrheal fecal specimens, total 1659, were collected from AGE patients with age range 0–84 from ten local hospitals in Seoul during 2014–2016 (Fig. 1). The samples were weekly collected and pretreated on arrival to test by qRT-PCR using PowerCheck™ Norovirus GI/GII (Kogene, Korea). For phylogenetic analysis, partial regions of VP1 for GI and GII genotypes were amplified by RT-PCR and semi-nested RT-PCR with the primers described in Table 1 [13] using the HyQ™ One-step RT-PCR kit (SNC, Korea). To apply one-step RT-PCR, the specific primer pairs (GI-F1M/GI-R1M and GII-F1M/GII-R1M) targeting VP1 in ORF2 were applied. Semi-nested PCR was performed using the one-step RT-PCR product (2 μl) and the primers (GI-F2/GI-R1M and GII-F3/GII-R1M). Before we request DNA sequencing in Macrogen, Inc. (Seoul, Korea), the RT-PCR products were purified using the QIAquick Gel Extraction Kit (Qiagen, Hilden, Germany). Analysis of nucleotide sequences was carried out by Macrogen using the Big Dye Dideoxy cycle sequencing kit and the ABI PRISM 3730XL Analyzer (Applied Biosystem, USA). The diagnostic practice for NoV detection was conducted according to the guidelines of the KCDC [12] and the manufacturer’s instructions.

**Table 1:** Primer sequences for norovirus detection

Virus	Primer	Sequence (5′ → 3′)	Position (nt)	Size (bp)	References
Norovirus GI^a^	GI-F1M	CTGCCCGAATTYGTAAATGATGAT	5336–5359	314	[13]
	GI-R1M	CCAACCCARCCATTRTACATYTG	5643–5665		
	GI-F2	ATGATGATGGCGTCTAAGGACGC	5352–5374		
Norovirus GII^a^	GII-F1M	GGGAGGGCGATCGCAATCT	5049–5067	313	[13]
	GII-R1M	CCRCCIGCATRICCRTTRTACAT	5367–5389		
	GII-F3	TTGTGAATGAAGATGGCGTCGA	5077–5100		

^a^Carried out by RT-PCR and semi-nested PCR

In our present study, total 4073 diarrheal specimens (1659 sporadic, 2414 outbreak) of AGE patients could cover in part the population in Seoul to represent NoV trend in Korea. In addition, we have expanded the study period of Seoul NoV epidemicity from the 3 year (2014–2016) to 10 year (2007–2016) by reanalyzing the unreported-NoV surveillance data (Fig. 3).

**Fig. 2:**
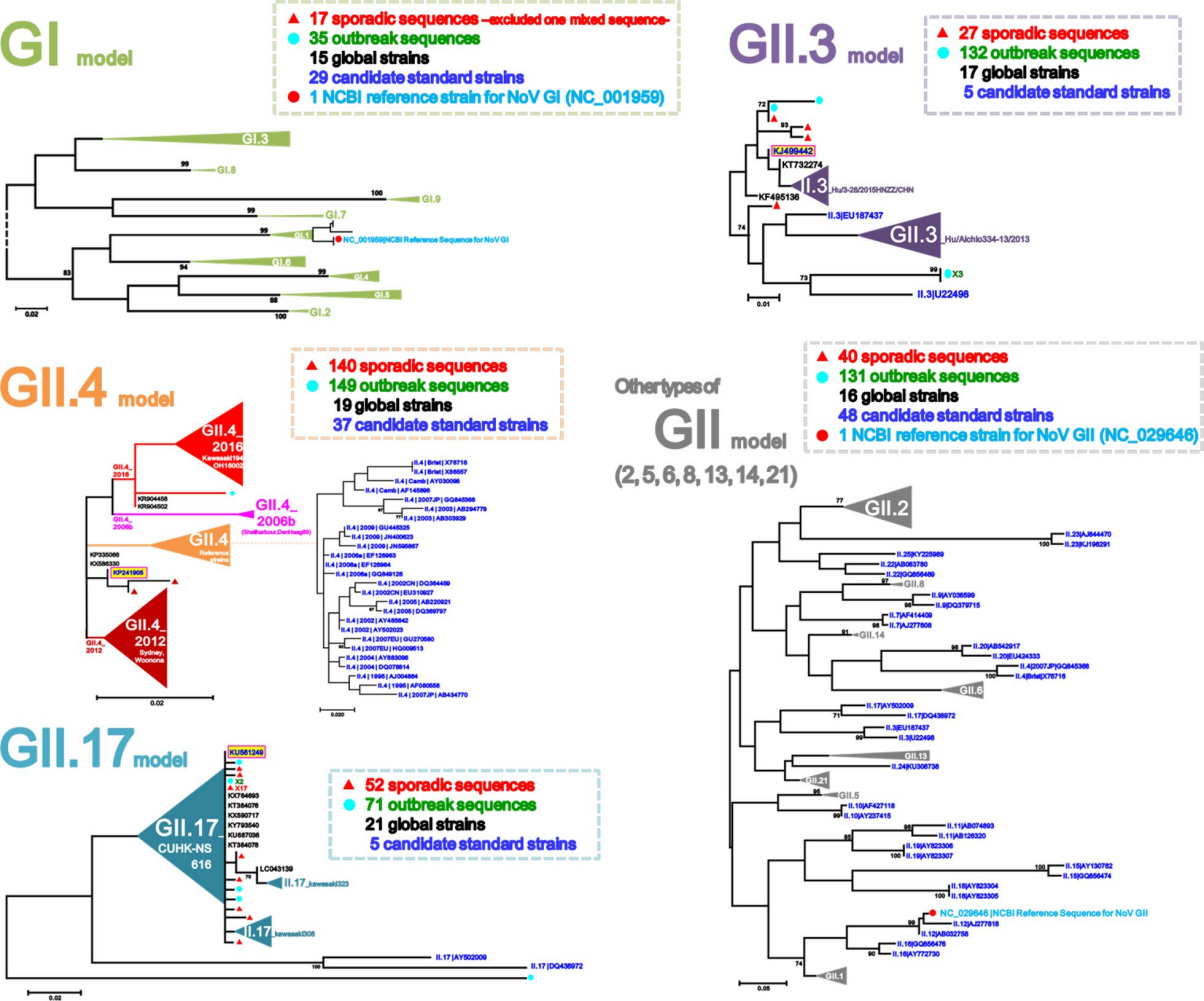
Model scheme of the phylogenetic trees of norovirus (GI/GII.4/GII.17/GII.3/other types of GII) in Seoul. The signs of solid triangle in red and the solid circles in blue indicate the data from surveillance and outbreak, respectively. Characters in blue indicate the candidate standard strains recommended in NoroNet and GenBank for NoV genotyping and sub-clustering. Characters in black indicate global strains obtained from GenBank

**Fig. 3:**
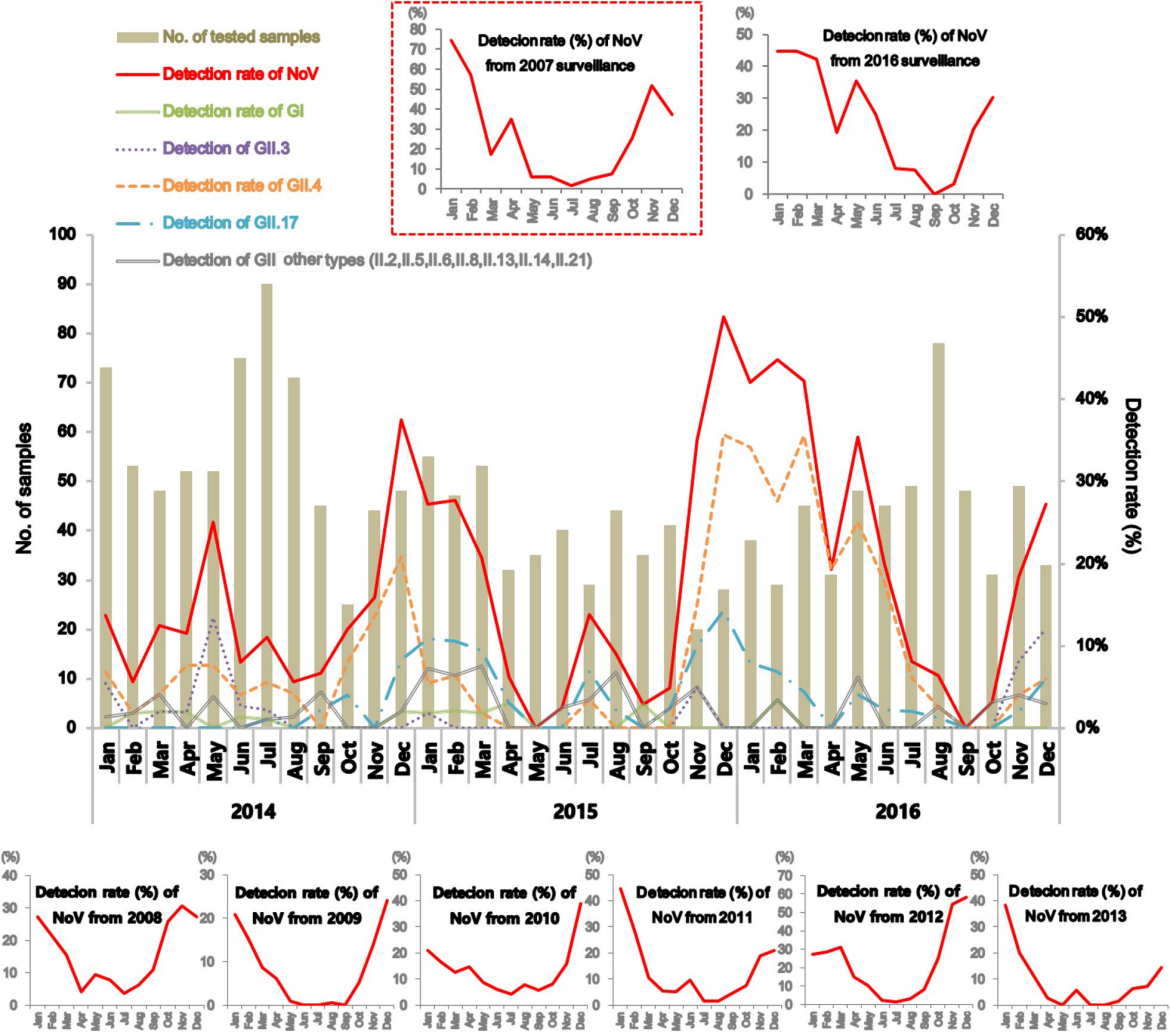
Norovirus genotypes detected in the sporadic AGE collected from the surveillance hospitals during 2014–2016. The solid bar indicates number of tested samples and the lines indicate detection rate of norovirus genotypes. The maximum detection rate is in December and the lowest is in September. Dotted box in red indicates the norovirus epidemic curve from 2007 surveillance

To detect sporadic strains developed into outbreaks in the Seoul NoV-surveillance system, 518 strains out of 2414 AGE patients obtained from outbreaks in Seoul during January 2014–June 2017 were compared with 276 strains out of 1659 AGE patients obtained from the surveillance in January 2014–December 2016. To examine the phylogenetic relationship with reference strains, 126 candidate standard strains (Additional file 1: Table S1) and 88 global strains (selected only as highly similar to our strains among updated NCBI sequences in each genotype) of NoV GI and GII were collected from NoroNet and GenBank, and then phylogenetically analyzed above-mentioned 794 (518 + 276) strains in the five NoV models, GI/GII.4/GII.17/GII.3/other types of GII (Fig. 2). To investigate presence or absence of unreported-NoV epidemic curves, we reanalyzed the data [14, 15] that had been previously obtained from the Seoul NoV-surveillance system in 2007–2013 (Fig. 3).

### Phylogenetic analysis

With rapid accumulation of huge norovirus sequence data [16] and an attempt to establish a unified norovirus classification in the CDC [17], the complete VP1 was replaced by the partial nucleotide sequence of a highly-variable N-terminal region in the VP1 (277-nucleotides). Therefore, the partial VP1 capsid region in ORF2 has routinely been used since 2006 to investigate genotyping and presence of norovirus variants by using a web-based automated typing tool [18]. For convenience of analysis, the primer sets [13] targeting a partial VP1 region have been recommended for PCR cloning and sequencing in the National Norovirus Surveillance Guideline in Korea [12]. In the five phylogenetic trees (GI/GII.4/GII.17/GII.3/other types of GII), 276 sporadic sequences and 518 outbreaks were aligned with each candidate standard strains (Additional file 1: Table S1) and additional global strains (Fig. 2). Phylogenetic analysis was performed using MEGA7.0 (Fig. 2) based on the partial VP1 sequences (289 nucleotides at the 5360–5648 with reference to NC_001959 for GI, and 279–293 nucleotides from the 5085 to 5363–5377 with reference to NC_029646 for GII). All aligned sequences were trimmed at the above-mentioned nucleotide positions as that of each NoV GI and GII strain. Maximum Likelihood phylogenetic trees constructed in MEGA 7.0 program were inferred from the 1000 replicates based on the Tamura-Nei model with a bootstrap consensus tree, and the bootstrap values were given above 70%.

## Results

### Prevalence of NoV

NoV was detected in the 271 specimens out of 1659 (16.34%) and the annual detection rate was increased by 7.44% from 13.17% in 2014 to 20.61% in 2016; presence in 89 samples among 676 in 2014, 74 among 459 samples in 2015, and 108 among 524 samples in 2016. In monthly surveillance over the 3 years (2014–2016), NoV was detected almost all year around except in May, 2015 and September, 2016 (Fig. 3). Genotyping was performed with 271 NoV-positive fecal specimens and found that prevalence of GI (12/271 positives) was 4.43%, that of GII (253/271 positives) was 93.36%, and GI and GII combined was 2.21% (6/271 positives) (Table 2). The most prevalent strain of NoV was found to be GII.4; prevalence of GII.4 was 50.92% (138/271 positives), GII.17 was 18.08% (49/271 positives), GII.3 was 9.96% (27/271 positives), GII.6 was 5.54% (15/271 positives), GII.21 was 4.06% (11/271 positives), GII.13 was 2.58% (7/271 positives), GII.2 was 1.48% (4/271 positives), and the rarely detected GII.5, GII.8, and GII.14 was positive only one fecal specimen out of 271 (0.37%) (Fig. 3 and Table 2).

**Table 2:** Genotypes of sporadic noroviruses and number of samples identified from the Seoul surveillance test during 2014–2016

Enteric virus	Genotype	No. of samples	Genotype	No. of samples	Total
Norovirus GI (no. of single GI genotype)	GI.1	0	GI.5	2	12
	GI.2	1	GI.6	1	
	GI.3	3	GI.7	1	
	GI.4	3	Mixed	1	
Norovirus GII (no. of single GII genotype)	GII.2	4	GII.8	1	253
	GII.3	27	GII.13	7	
	GII.4	138	GII.14	1	
	GII.5	1	GII.17	49	
	GII.6	15	GII.21	10	
Norovirus GII.4 (no. of single GII4 genotype)	GII.4|2006b	1	GII.4|2016	70	138
	GII.4|2012	67	(GI & GII.4|2012)	(2)	
Norovirus GI & GII (no. of GI and GII combination genotype)	GI.1 & GII.17	1	GI.5 & GII.17	1	6
	GI.3 & GII.17	1	GI.5 & GII.21	1	
	GI.4 & GII.4	1	GI.6 & GII.4	1	

### Monthly distribution of NoV epidemicity

Monthly prevalence trend of the NoV genotypes was variable (Fig. 3 and Table 3). To compare the previous data obtained from the Seoul NoV-surveillance during the past 7 years with our present study, the highest and the lowest NoV frequency were recorded, respectively, as 35.60% (366/1028 samples) in January and 2.44% (20/818 samples) in July in the 2007–2011 surveillance [14], and 37.78% in December (68/180 samples) and 0.86% in July (1/116 samples) in the 2012–2013 surveillance [15]. In the 2014–2016 surveillance, the highest frequency was found in December (37.61%; 41/109 samples) and the lowest was in September (3.13%; 4/128 samples). The overall epidemicity of the Seoul NoV-surveillance recorded in the 2008–2013 and the 2014–2015 had been found as the “W-shape” and the typical “U-shape” curve, respectively, with seasonal variability, however, the surveillance data detected in the 2007 (dotted box in Fig. 3) and the 2016 revealed a novel “right-sided W-shape” rather than the typical “W (or U)-shape” curve. In the novel “right-sided W-shape” curve in 2016, the major genotype was found to be GII.4 that was the same as the main genotype occurred in the 2014 Seoul NoV-surveillance. Compared to the previous data, the abnormally high activity of the NoV found in winter of 2015–2016 and the high peak found in spring of 2016 could be caused the novel “right-sided W-shape” curve recorded in the 2016 Seoul NoV-surveillance. The main genotype detected in the 2015 surveillance was identified as GII.17 that may cause the typical “W-shape” curve (Fig. 3 and Table 3).

**Table 3:** Distribution of norovirus genotypes detected by surveillance test during 2014–2016

Month	No. of strains of norovirus GI and GII from Seoul in 2014–2016													
	GI	I&II	II.2	II.3	II.4	II.5	II.6	II.8	II.13	II.14	II.17	II.21	No. of NoVs	No. of tested
January	1	–	–	5	21	–	3	–	2	–	9	–	41	166
February	3	2	–	–	12	–	–	1	3	–	7	1	29	129
March	2	1	1	1	19	–	–	–	2	–	7	3	36	146
April	2	–	–	1	1	–	–	–	–	–	1	–	14	115
May	–	–	–	7	16	1	3	–	–	–	2	1	30	135
June	1	–	–	2	11	–	–	–	–	–	1	1	16	160
July	1	–	–	2	9	–	1	–	–	–	4	1	18	168
August	–	–	1	–	5	–	1	–	–	1	2	3	13	193
September	1	–	–	–	–	–	2	–	–	–	1	–	4	128
October	–	–	–	–	2	–	2	–	–	–	2	–	6	97
November	–	1	1	5	11	–	2	–	–	–	3	–	23	113
December	1	2	1	4	22	–	1	–	–	–	1	–	41	109
Total	12	6	4	27	138	1	15	1	7	1	49	10	271	1659

### Norovirus phylogenetic analysis: GI, GII.4, GII.17, GII.3 and other types of GII (II.2/5/6/8/13/14/21)

**Fig. 4:**
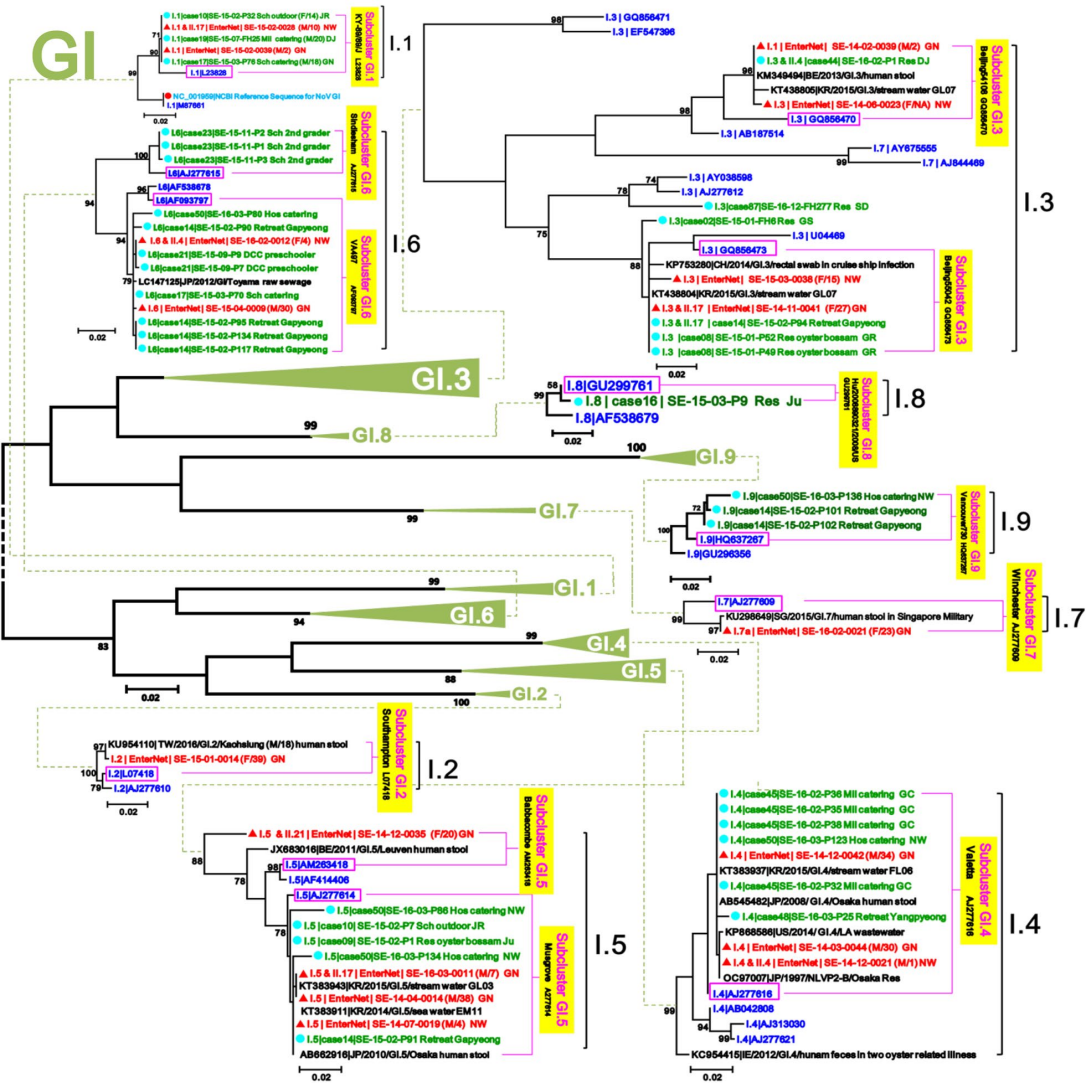
Maximum Likelihood phylogenetic trees of partial VP1 in norovirus GI. The signs of solid triangle in red and the solid circles in blue indicate the data from surveillance and outbreak, respectively. Characters in blue indicate the candidate standard strains recommended in NoroNet and GenBank for NoV genotyping and sub-clustering. Characters in black indicate global strains obtained from GenBank. Phylogenetic tree of GI showing the variable strains of norovirus detected in the Seoul surveillance during 2014–2016

In the GI phylogenetic tree, the 17 sporadic sequences presented a great diversity of seven different clusters (I.1/I.2/I.3/I.4/I.5/I.6/I.7). The dominant genotypes in the sporadic GI were GI.3, GI.5, and GI.4. Six combinations of GI and GII (I.1_II.17/I.3_II.17/I.4_II.4/I.5_II.17/I.5_II.21/I.6_II.4) were detected in the GI surveillance. In the GI.3, GI.5 and GI.6 clusters, two or four sporadic sequences were sub-divided into two sub-clusters; GI.3|Beijing55042/54108, GI.5|Musgrove/Babbacombe and GI.6|VA497/Sindlesham. In GI.1 and GI.4 clusters, two and three sporadic sequences were sub-divided into only one sub-cluster (GI.1|KY-89/89/J and GI.4|Valetta). I.2 and I.7 were sub-divided into one sub-cluster (I.2|Southampton and I.7|Winchester) with only one sporadic sequence. The I.8 and I.9 clusters included one and three outbreaks, but not sporadic one (Figs. 3 and 4).

**Fig. 5:**
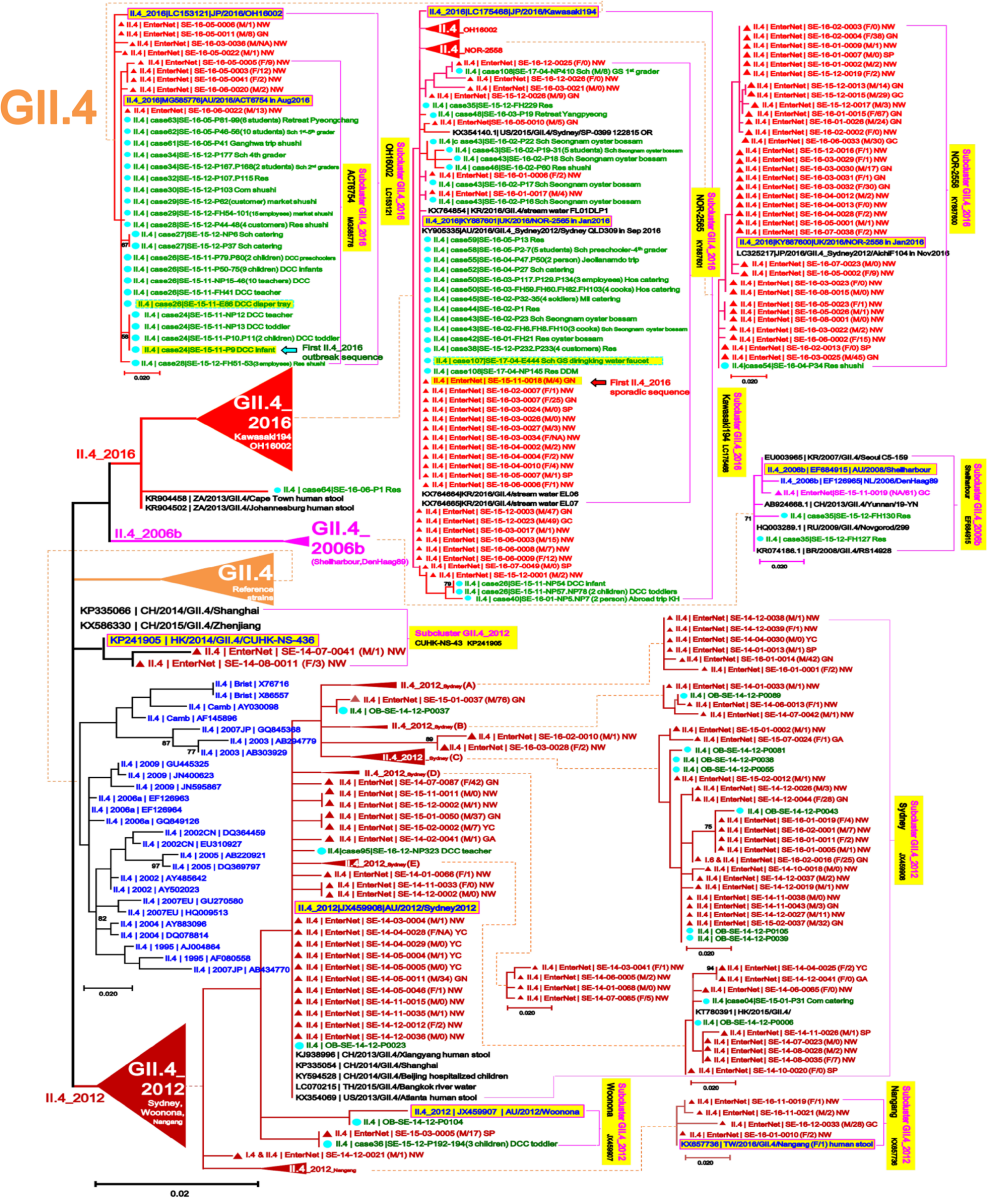
Maximum Likelihood phylogenetic trees of partial VP1 in norovirus GII.4. The signs of solid triangle in red and the solid circles in blue indicate the data from surveillance and outbreak, respectively. Characters in blue indicate the candidate standard strains recommended in NoroNet and GenBank for NoV genotyping and sub-clustering. Characters in black indicate global strains obtained from GenBank. Phylogenetic tree of GII.4, the most prevalent genotype, detected in the Seoul surveillance during 2014–2016. Note the characteristically divided GII.4 into GII.4_2016 and GII.4_2012 (in red and orange cluster arrow shapes, respectively)

In the most prevalent GII.4 phylogenetic tree, the 140 sporadic sequences were sub-divided into three sub-clusters (GII.4|2006, GII.4|2012 and GII.4|2016). Out of the 140 sporadic sequences, only one sporadic sequence was found in the GII.4|2006 sub-cluster. In the remaining 139 sporadic sequences, 69 sequences were clustered into the GII.4|2012 sub-cluster, and the last 70 sequences were clustered into GII.4|2016 sub-cluster. The three GII.4 sub-clusters showed a significant difference in their collection time and the development of outbreak; GII.4|2016 sporadic NoV was consistently observed after November 2015 and successfully developed into outbreak at the same time. We assumed that there was no preparation time between the sporadic occurrence and the outbreak explosion caused by the GII.4|2016_Kawasaki194 sub-lineages (ACT6754/NOR-2565/NOR-2558). However, the 69 sporadic NoV in GII.4|2012 (GII.4|2012_Sydney/Kawasaki194) frequently detected before November 2015 and failed to develop outbreak during the whole study period. Our phylogenetically analysis may demonstrate GII.4|2016_Kawasaki194 emergence very rapidly in Seoul since December 2015, which might form the novel “right-sided W-shape” curve (Figs. 3 and 5).

**Fig. 6:**
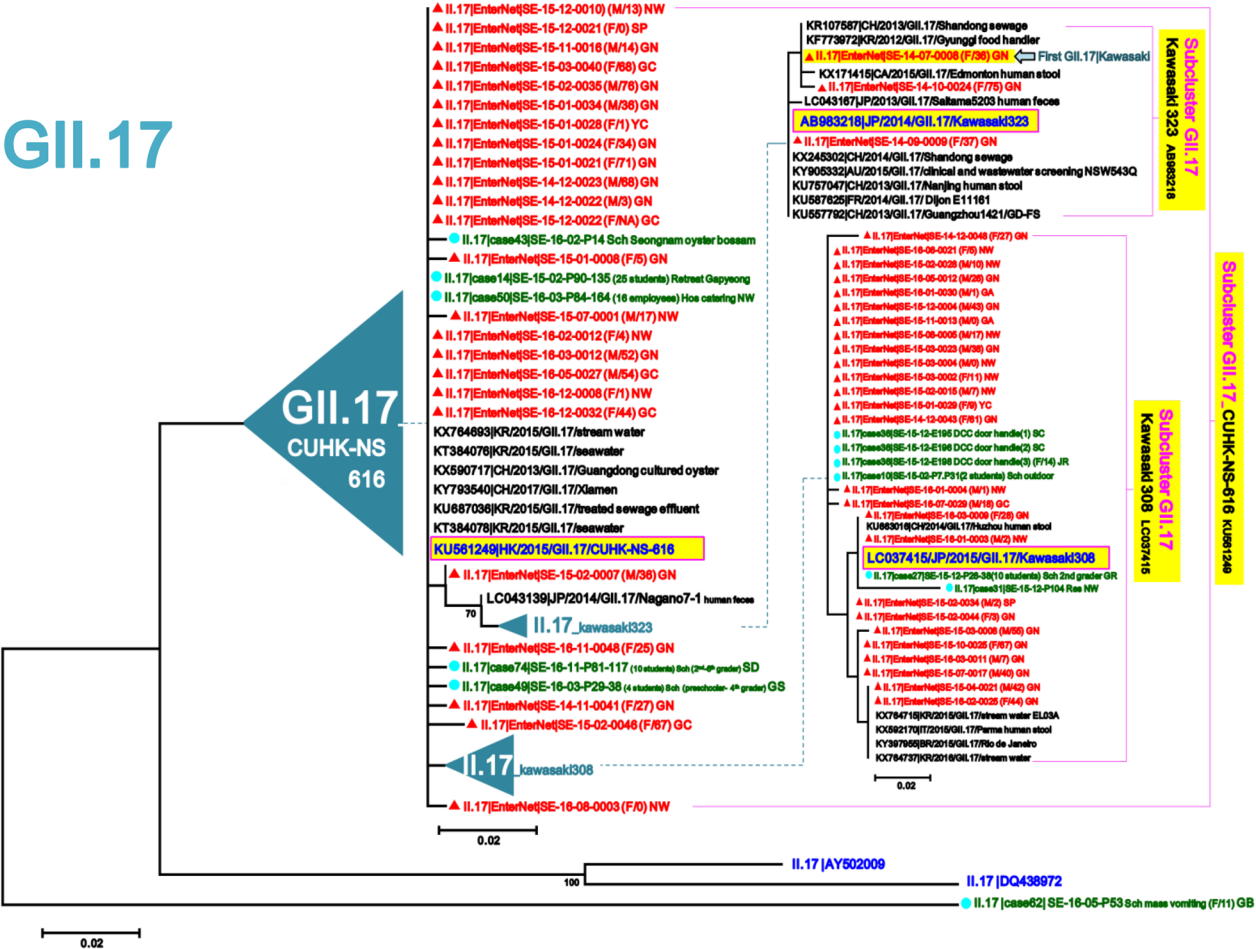
Maximum Likelihood phylogenetic trees of partial VP1 in norovirus GII.17. The signs of solid triangle in red and the solid circles in blue indicate the data from surveillance and outbreak, respectively. Characters in blue indicate the candidate standard strains recommended in NoroNet and GenBank for NoV genotyping and sub-clustering. Characters in black indicate global strains obtained from GenBank. Phylogenetic tree of GII.17, the second most prevalent genotype, detected in the Seoul surveillance during 2014–2016. Main GII.17|CUHK-NS-616 was further sub-divided into GII.17|Kawasaki308 and GII.17|Kawasaki323

In the GII.17 phylogenetic tree, the 52 sporadic sequences GII.17|CUHK-NS-616 (KU561249) were clustered into two sub-clusters (GII.17|Kawasaki308/Kawasaki323). The first sporadic GII.17 from our surveillance emerged as GII.17|Kawasaki323 in July 2014. Since we started surveillance in 2015, GII.17 was found to be the predominant genotype with GII.17|2014_Kawasaki lineage (CUHK-NS-616/Kawasaki308) for the first time, which replaced the previously dominant GII.4 genotype. Although occurrence of GII.17 continuously increased from December 2014 to March 2015, it could not overcome the outbreak explosion by GII.4|2016_Kawasaki194 in November 2015 (Figs. 3 and 6).

**Fig. 7:**
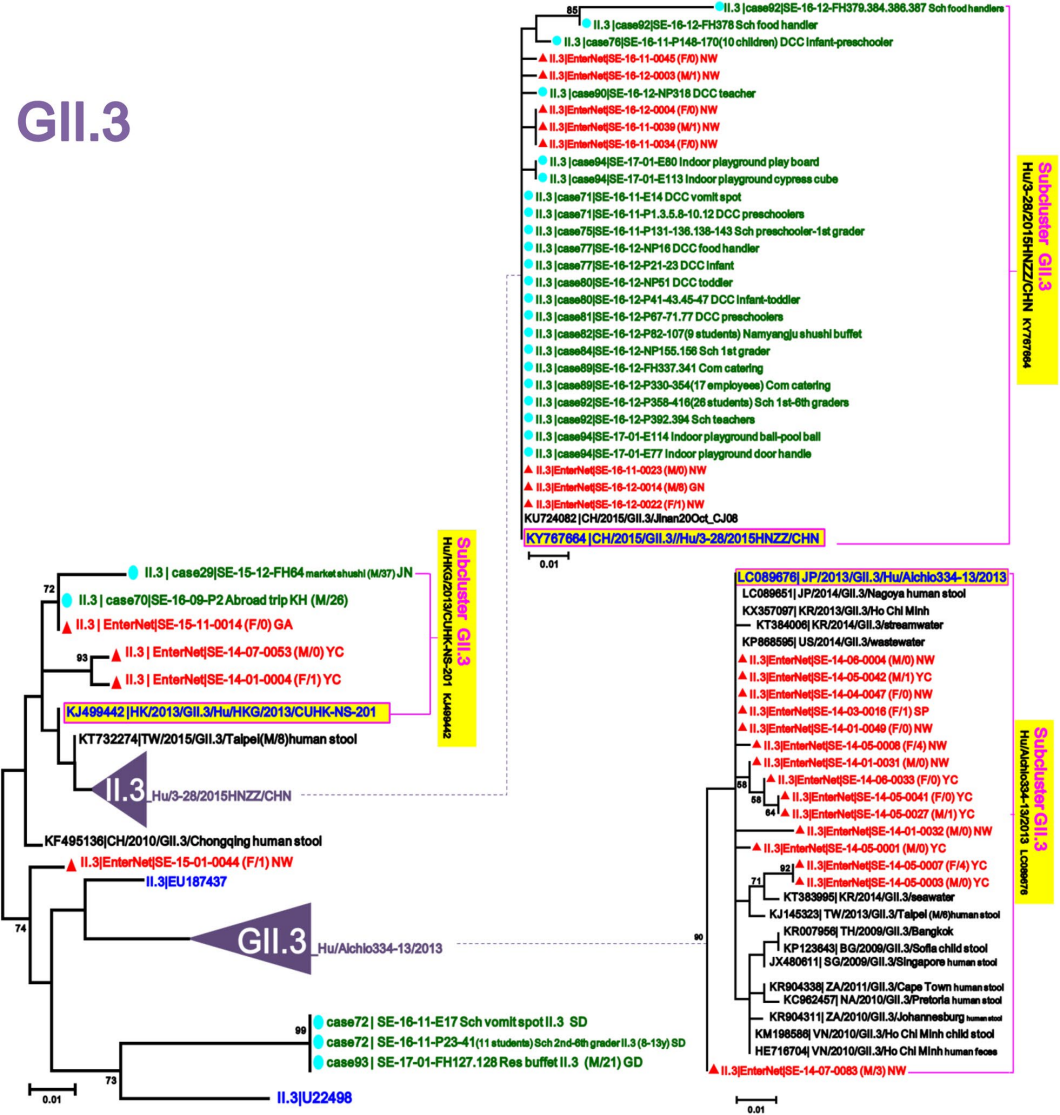
Maximum Likelihood phylogenetic trees of partial VP1 in norovirus GII.3. The signs of solid triangle in red and the solid circles in blue indicate the data from surveillance and outbreak, respectively. Characters in blue indicate the candidate standard strains recommended in NoroNet and GenBank for NoV genotyping and sub-clustering. Characters in black indicate global strains obtained from GenBank. Phylogenetic tree of GII.3, the third most prevalent genotype, detected in the Seoul surveillance during 2014–2016. GII.3|Hu/3-28/2015/HNZZ/CHN was detected from both outbreak and surveillance, however, GII.3|Hu/Aichio334-13/2013 was found only in surveillance test

In the GII.3 phylogenetic tree, the 26 sporadic sequences were clustered into three sub-clusters (GII.3|Hu/Aichio334-13/2013, GII.3|Hu/3-28/2015/HNZZ/CHN and GII.3|Hu/HKG/2013/CUHK-NS-201). In the phylogenetic tree, none of the sporadic GII.3 sequences presented any identical outbreak one, except in an overseas inflow. Although GII.3|Hu/Aichio334-13/2013 (LC089676) was the most prevalent GII.3 strain in the Seoul NoV-surveillance during 2014–2016, any of the 15 sporadic sequences failed to develop any outbreak. In GII.3|Hu/3-28/2015/HNZZ/CHN (KY767664) causing the GII.3 explosion in Seoul since November 2016, the first sequences from our surveillance and the outbreak emerged simultaneously in November 2016. We thought that there was no preparation time between the sporadic occurrence and the outbreak associated with the GII.3|Hu/3-28/2015/HNZZ/CHN strains (Figs. 3 and 7).

**Fig. 8:**
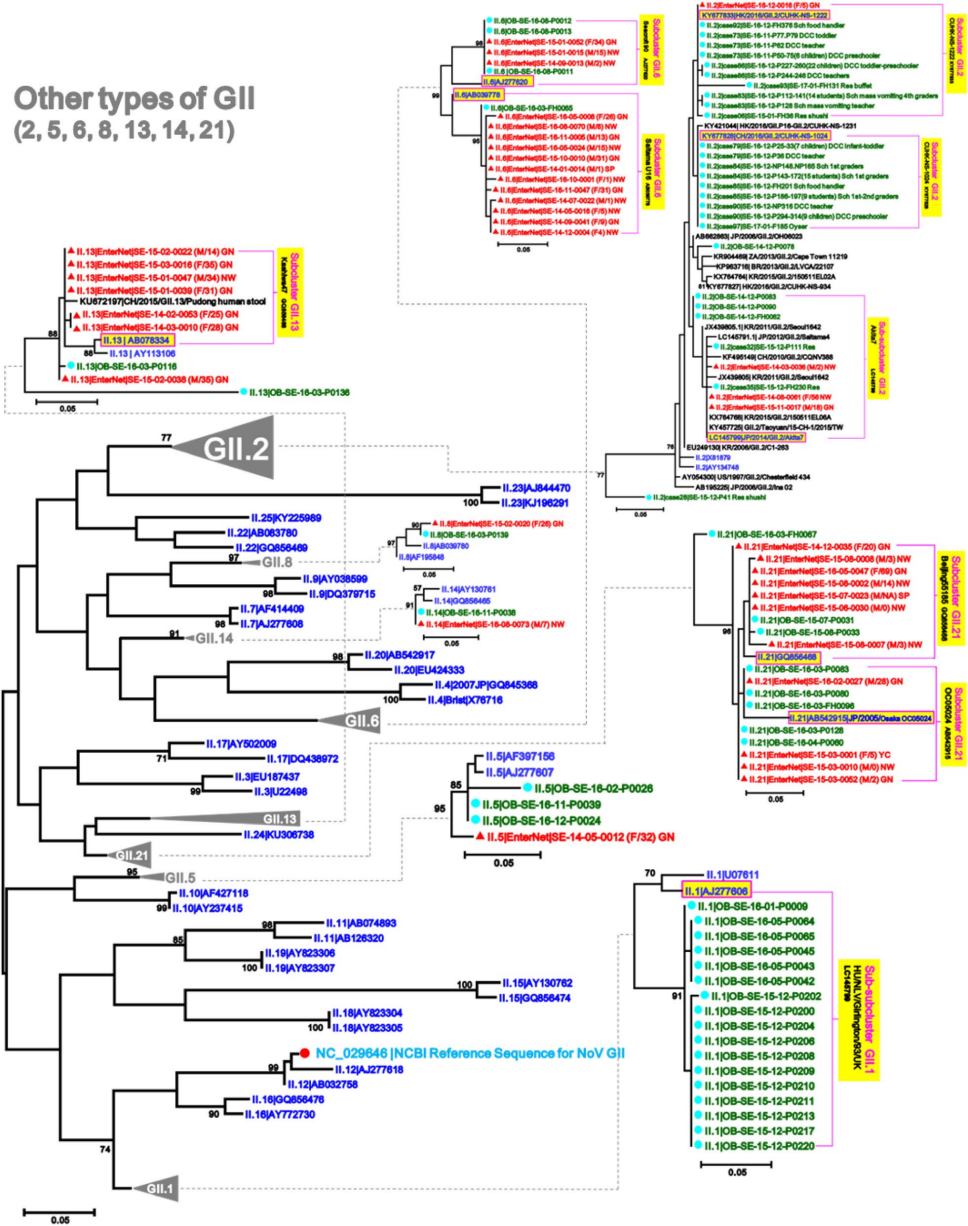
Maximum Likelihood phylogenetic trees of partial VP1 in other types of norovirus GII. The signs of solid triangle in red and the solid circles in blue indicate the data from surveillance and outbreak, respectively. Characters in blue indicate the candidate standard strains recommended in NoroNet and GenBank for NoV genotyping and sub-clustering. Characters in black indicate global strains obtained from GenBank. Phylogenetic tree of other types of GII (2/5/6/8/13/14/21) showing variable strains of norovirus detected in the Seoul surveillance during 2014–2016

In the other types of GII phylogenetic tree, the 40 sporadic sequences were clustered into seven GII clusters (2/5/6/8/13/14/21) and seven GII sub-clusters. In the other types of GII phylogenetic tree, about eight sporadic strains developed into nine outbreaks. In the GII.6 cluster, 15 sporadic sequences and four outbreak ones were sub-divided into two sub-clusters (GII.6|Seacroft90/SaitamaU16). In the GII.2 cluster, four sporadic sequences and 95 outbreaks were sub-divided into three sub-clusters (GII.2|Akita7/CUHK-NS-1222/CUHK-NS-1224). 52 GII.2|CUHK-NS-1222 (KY677833) outbreak strains and 37 GII.2|CUHK-NS-1024 (KY677828) outbreak strains caused the GII.2 explosion in Seoul since November 2016. In the GII.21 cluster, 11 sporadic sequences were sub-divided into two sub-clusters (GII.21|Beijing-55185/OC05024). The clusters GII.5, GII.8, and GII.14 were further divided into one sub-cluster (GII.5|Hillingdon90, GII.8|Amsterdam, and GII.14|Beijing) with only one sporadic sequence belonging to a sub-cluster. Although the GII.1 cluster and the GII.2|CUHK-NS-1024 sub-cluster included 17 and 37 outbreaks, respectively, they did not include any sporadic sequences (Figs. 3 and 8).

## Discussion

To characterize phylogenetic epidemiology of NoV strains circulating in Seoul, we first conducted an extensive phylogenetic analysis based on partial VP1 sequences of total 1008 NoV (794 NoV positives from 4073 AGE specimens and 214 global references from NoroNet and GenBank). This extensive phylogenetic analysis revealed the monthly prevalence and distribution of NoV strains widespread in Seoul and their phylogenetic relationships with global reference strains. Through the five phylogenetic trees (GI/GII.4/GII.17/GII.3/other types of GII) obtained from the NoV-surveillance in 2014–2016, we could track emergences of the new strains and determine the trends of circulating strains in Seoul (Figs. 4, 5, 6, 7, 8).

In the GI phylogenetic tree, the three sporadic sequences (GI.4|Valetta, GI.3|Beijing55042 and GI.5|Musgrove) found in 2014 were identical to the nine outbreaks in 2015–2016. They were also identical to the global strains (AB545482 and KT383937 in GI.4|Valetta, KT438804 in GI.3|Beijing55042, and AB662916 and KT383943 in GI.5|Musgrove) collected from human stools of Japan in 2008 and 2010, and from sea-water and stream-waters of Korea in 2014 and 2015 (Fig. 4). The data can suggest that the origin of Seoul sporadic GI strains may come from Japan.

In the three major phylogenetic trees of GII.4, GII.17 and GII.3, all the strains reported in the present study were the same as the global strains reported in NoroNet and GenBank. In the GII.17 phylogenetic tree, the first occurrence was detected as GII.17|Kawasaki323 (AB983218) in July 2014, which was identical to the new GII.P17-GII.17 reported in March 2014 in Japan [19]. The new GII.17 was 96% homologous in the amino acid sequences with the GII.17 strain reported in Korea by KCDC [20]. Since first emergence of GII.17|Kawasaki323, GII.17|CUHK-NS-616 and GII.17|Kawasaki308 sharply increased during the winter in 2014–2015, and the GII.17 became the first dominant genotype in Seoul in January 2015 (Figs. 3 and 6). This epidemicity was well accordant with the global trend of the new GII.17 (GII.17|Kawasaki). According to the previous report by KCDC, the GII.17, previously considered as a minor type in Korea, is the predominant NoV since December 2014 [20]. During the winter in 2014–2015, the new GII.17 also emerged and became the predominant genotype in Japan [19], several major cities in mainland China [21, 22], and HongKong [23]. The new GII.P17-GII.17 was also detected sporadically outside of Asia such as Italy, Romania, and the United States [24–26]. In our previous surveillance data during 2008–2011, 15 sporadic GII.17 were detected three times; 13 in March 2008, one in August 2008 and one in November 2010 [14]. The sporadic GII.17 in Seoul might be different from the past and current prevalent strains. The GII.17 detected in this study was presumed to be the new GII.17 that caused a pandemic since the winter of 2014.

In our sporadic data, GII.4 was still the most prevalent genotype (50.92%, 138/271 positives) during the Seoul NoV-surveillance in 2014–2016 (Tables 2, 3 and Figs. 3, 5). In the NoV GII.4 phylogenetic tree, all the sporadic GII.4 sequences were tightly clustered together, and largely sub-divided into two sub-clusters (GII.4|2012 and GII.4|2016) (Figs. 2 and 5). These sub-clusters showed distinct difference from the sporadic strains to outbreaks and seasonal epidemics. According to the previous report about GII.4|2012_Sydney, it was the most frequently found sub-genotype (60.4%) during November 2012 and January 2013 in Korea [27]. GII.4|2012_Sydney was still the most frequently found sub-genotype (51.69%, 46/89 positives) of the Seoul NoV-surveillance in 2014 (Fig. 5). Since November 2015, GII.4|2012 was replaced internally by novel GII.4|2016_Kawasaki194 (NOR-2565/NOR-2558/OH16002) that was the variants of GII.4|2012_Sydney (JX459908). The sharply increased sporadic NoV in the winter of 2015 (Fig. 3) was mainly caused by II.4|2016_Kawasaki194 (LC175468) that was first detected from AGE patients in Kawasaki City in 2016 [28]. Since its first detection, GII.4|2016_Kawasaki194 spread very rapidly and caused sporadic NoV explosion, which raised the average NoV detecion rate from 13.17% (89/676 samples in 2014) to 20.61% (108/524 samples in 2016) during the present study period. Due to unusually high NoV activity, seasonal epidemic curve in 2016 was skewed to the novel “right-sided W-shaped” curve (Fig. 3). By reanalyzing the Seoul NoV-surveillance data in 2007–2013, we found that the epidemic curve in 2007 was similar with our novel “right-sided W-shape” curve in 2016 (dotted box in Fig. 3). Additional evolution studies are required to investigate why the estimated GII.4|2006b and GII.4|2016_Kawasaki194 strains spreaded more rapidly and caused heavy explosions in Seoul in 2007 and 2016 compared to other pandemic strains. In the II.4|2016_Kawasaki194 sub-cluster, our 11 sporadic sequences were identical to four global strains (KY887601, KY905335, KX764665 and KX764664), which were collected from human stools and stream waters in January and September 2016. Above-mentioned four global strains and the II.4|2016_Kawasaki194 strain were all reported as GII.P16-GII.4 Sydney2012 recombinants [29, 30]. The first appearance of the Seoul sporadic Kawasaki194 strain was in November 2015, which is at least a few months ahead of identical global strains and the II.4|2016_Kawasaki194 candidate standard strain.

In our outbreak data, GII.3 and GII.2 exploded and caused huge outbreaks during the winter of 2016 (Figs. 7 and 8). Although sporadic GII.3 became the predominant genotype in last quarter of 2016 from the Seoul NoV-surveillance (Figs. 3 and 7), sporadic GII.2 remains low until December 2016 (Figs. 3 and 8). Although GII.3|Hu/3-28/2015/HNZZ/CHN strain caused heavy outbreaks in Seoul since November 2016, the most dominant strain in the Seoul NoV-surveillance was not GII.3|Hu/3-28/2015/HNZZ/CHN but GII.3|Hu/Aichio334-13/2013 (Fig. 7). In the GII.3 phylogenetic analysis, we also observed that GII.3|Hu/Aichio334-13/2013, the dominant strain in 2014, could not develop outbreak in Seoul, whereas GII.3|Hu/3-28/2015/HNZZ/CHN rapidly developed outbreak. We, here, suggest that the sporadic GII.3|Hu/3-28/2015/HNZZ/CHN might be a GII.P16-GII.3 variant. Although sporadic GII.2 and GII.6 were detected at low prevalence during the whole study period, they are important genotypes prevalent in worldwide and should be carefully monitored [31, 32].

### Limitation

We agree with the presence of several limitations in the present study to determine the geographical distribution and mechanism of the NoV outbreak. However, this study was abided by the K-CaliciNet and the national norovirus surveillance guidelines [12]. The followings may explain potential reasons of our present limitation; First, present analysis of target sequences was confined to the partial VP1 region. Although it is not long enough to detect whole genome of NoV, the VP1 was designated as the NoV surveillance target sequences based on its high variability in sequences and efficiency. The sequencing of partial VP1 also allows large quantities of NoV to be genotyped economically with epidemiologic trends. A novel NoV lineage containing the GII.P16 polymerase and pandemic GII.4 Sydney and other GII capsid were recently detected in Asia and Europe during the winter in 2016 to 2017 [29]. To examine NoV evolutions in recombination and surface-exposed antigenic regions, future study should be focused on exploring the large target sequences covering RdRp and complete VP1 region. Second, the 10 hospitals employed for the present study were not evenly distributed in Seoul (three out of 10 hospitals were localized in one administrative district-Gu) (Fig. 1). Although we could track new strains through phylogenetic analysis with outbreaks and global strains, it was insufficient to cover the detailed NoV transmission routes. Lastly, our sample collection was limited to the patients with symptomatic infection, however, over 30% of NoV infection is asymptomatic with shedding virus [33].

## Conclusions

During 2014–2016, we determined 17 NoV genotypes and their sub-genotypes widespread in Seoul. By the first extensive phylogenetic characterization of 1008 specimens, we could track the emergence of new NoV strains that is able to cause massive outbreak or sporadic AGE infection globally. Most of them were found to be the novel variants of three major genotypes (GII.4, GII.17 and GII.3). Main epidemiologic event in the 2014–2016 Seoul NoV surveillance was continuous emergences of novel NoV strains of GII.17 (GII.17|2014_Kawasaki lineages in 2014), GII.4 (GII.4|2016_Kawasaki194 lineages in 2015), and GII.3 (GII.3|Hu/3-28/2015/HNZZ/CHN in 2016). Our results demonstrate that emergent GII.4|2016_Kawasaki194 lineages spread throughout Seoul very rapidly and caused unusually heavy explosions, especially in Seoul in 2016 compared to other two pandemic strains (GII.4|Sydney and GII.17|Kawasaki).

By analyzing the development from sporadic strains to outbreaks in various phylogenetic trees, we can show distinctly different patterns depending on each NoV lineages. Our report has an important implication in the understanding NoV incidence and developing a treatment vaccine against NoV.

## Additional file

**Additional file 1: Table S1.** Candidate standard strains for genotyping and sub-clustering.

## Abbreviations

AGE: acute gastroenteritis
NoV: norovirus
qRT-PCR: real-time reverse transcription polymerase chain reaction
RT-PCR: reverse transcription polymerase chain reaction
ORF: open reading frame
DB: Dobong
DDM: Dongdaemun
DJ: Dongjak
EP: Eunpyeong
GA: Gwanak
GB: Gangbuk
GC: Geumcheon
GD: Gangdong
GJ: Gangjin
GN: Gangnam
GR: Guro
GS: Gangseo
JN: Jungnang
Ju: Jung
JR: Jongno
MP: Mapo
NW: Nowon
SB: Seongbuk
SC: Seocho
SD: Seongdong
SDM: Seodaemun
SP: Songpa
YDP: Yeongdeungpo
YC: Yangcheon
YS: Yongsan
AU: Australia
BE: Belgium
BR: Brazil
CA: Canada
CH: China
HK: HongKong
Fin: Finland
IE: Ireland
IT: Italy
JP: Japan
KH: Cambodia
KR: Korea
NL: Netherlands
SG: Singapore
TH: Thailand
TW: Taiwan
UK: United Kingdom
US: United States
VN: Vietnam
ZA: South Africa
DCC: day-care center
Hos: hospital
Mil: military base
Res: restaurant
Sch: school
