# Supplementary material for: Phylogenetic characterization of norovirus strains detected from sporadic gastroenteritis in Seoul during 2014–2016
Source: Gut Pathog. 2018 Aug 27;10:36. doi: 10.1186/s13099-018-0263-8 (PMC6112129; doi:10.1186/s13099-018-0263-8)
Supplement: Supplementary file 1 — Additional file 1: Table S1. Candidate standard strains for genotyping and sub-clustering. [file 13099_2018_263_MOESM1_ESM.docx]

**Table S1. Candidate standard strains for genotyping and sub-clustering.**

| **Genotypes GenBank accession No.** | | | **Genotypes GenBank accession No.** | | |
| --- | --- | --- | --- | --- | --- |
| **Norovirus Genotyping** | **GI.1**  **GI.2**  **GI.3**  **GI.4**  **GI.5**  **GI.6**  **GI.7**  **GI.8**  **GI.9**  **GII.1**  **GII.2**  **GII.3**  **GII.4**  **GII.5**  **GII.6**  **GII.7**  **GII.8**  **GII.9**  **GII.10**  **GII.11**  **GII.12**  **GII.13**  **GII.14**  **GII.15**  **GII.16**  **GII.17**  **GII.18**  **GII.19**  **GII.20**  **GII.21**  **GII.22**  **GII.23**  **GII.24**  **GII.25** | **L23828, M87661**  **AJ277610, L07418**  **AB187514, AJ277612,**  **AY038598, EF547396, GQ856470,GQ856471,**  **GQ856473, U04469**  **AB042808, AJ277616,**  **AJ277621, AJ313030**  **AF414406, AJ277614,**  **AM263418**  **AF093797, AF538678,**  **AJ277615**  **AJ277609, AJ844469,**  **AY675555**  **AF538679, GU299761**  **GU296356, HQ637267**  **AJ277606 , U07611**  **AY134748, X81879**  **EU187437, U02030,**  **Brist_X76716,**  **2007JP_ GQ845368**  **AF397156, AJ277607**  **AB039778, AJ277620**  **AF414409, AJ277608**  **AB039780, AF195848**  **AY038599, DQ379715**  **AF427118, AY237415**  **AB074893, AB126320**  **AB032758, AJ277618**  **AB078334, AY113106**  **AY130761, GQ856465**  **AY130762**  **AY502010, GQ856476**  **AY502009, DQ438972**  **AB983218_Kawasaki**  **AY823304, AY823305**  **AY823306, AY823307**  **AB542917, EU373815**  **AB083780, GQ856469**  **AJ844470, KJ196291**  **KU306738**  **KY225989** | **Norovirus Sub-clustering** | **GII.4**  **GII.17**  **GII.3**  **GI**  **GII** | **1995_AF080558,**  **1995_AJ004864**  **2002_AY485642,**  **2002_AY502023**  **2002CN_DQ364459,**  **2002CN_EU310927**  **2003_AB294779,**  **2003_AB303929**  **2004_AY883096,**  **2004_DQ078814**  **2005_AB220921,**  **2005_DQ369797**  **2006a_EF126963,**  **2006a_EF126964**  **2006b_EF126965,**  **2006b_EF684915**  **2006a_GQ849126,**  **2007EU_HQ009513**  **2007EU_GU270580,**  **2009_GU445325**  **2007JP_AB434770,**  **2007JP_GQ845368**  **2009_JN400623,**  **2009_JN595867**  **2012_JX459907, KX657736**  **2012_JX459908, KP241905**  **2016_LC153121, LC175468**  **2016_KY887600, KY887601**  **2016_MG585776**  **Brist_X76716,**  **Brist_X86557**  **Camb_AY030098,**  **Camb_AF145896**  **AB983218, AY502009,**  **DQ438972, KU561249**  **LC037415,**  **EU187437, U02030,**  **KJ499442, KY767664, LC089676**  **NC_001959.2**  **NC_029646.1** |
